# Improved ^13^C metabolic flux analysis in *Escherichia coli* metabolism: application of a high-resolution MS (GC–EI–QTOF) for comprehensive assessment of MS/MS fragments

**DOI:** 10.1093/jimb/kuad039

**Published:** 2023-11-13

**Authors:** Chris Richter, Eva Grafahrend-Belau, Jörg Ziegler, Manish L Raorane, Björn H Junker

**Affiliations:** Institute of Pharmacy, Martin Luther University Halle-Wittenberg, Hoher Weg 8, D-06120Halle (Saale), Germany; Institute of Pharmacy, Martin Luther University Halle-Wittenberg, Hoher Weg 8, D-06120Halle (Saale), Germany; Leibniz Institute of Plant Biochemistry, Weinberg 3, D-06120Halle (Saale), Germany; Institute of Pharmacy, Martin Luther University Halle-Wittenberg, Hoher Weg 8, D-06120Halle (Saale), Germany; Institute of Pharmacy, Martin Luther University Halle-Wittenberg, Hoher Weg 8, D-06120Halle (Saale), Germany

**Keywords:** GC–EI–MS/MS, Isotopomer, Metabolism, Stable isotope tracer, ^13^C-metabolic flux analysis

## Abstract

Gas chromatography–tandem mass spectrometry with electron ionization (GC–EI–MS/MS) provides rich information on stable-isotope labeling for ^13^C-metabolic flux analysis (^13^C-MFA). To pave the way for the routine application of tandem MS data for metabolic flux quantification, we aimed to compile a comprehensive library of GC–EI–MS/MS fragments of *tert*-butyldimethylsilyl (TBDMS) derivatized proteinogenic amino acids. First, we established an analytical workflow that combines high-resolution gas chromatography-quadrupole time-of-flight mass spectrometry and fully ^13^C-labeled biomass to identify and structurally elucidate tandem MS amino acid fragments. Application of the high-mass accuracy MS procedure resulted into the identification of 129 validated precursor–product ion pairs of 13 amino acids with 30 fragments being accepted for ^13^C-MFA. The practical benefit of the novel tandem MS data was demonstrated by a proof–of–concept study, which confirmed the importance of the compiled library for high-resolution ^13^C-MFA.

**One sentence summary:**

An analytical workflow that combines high-resolution mass spectrometry (MS) and fully ^13^C-labeled biomass to identify and structurally elucidate tandem MS amino acid fragments, which provide positional information and therefore offering significant advantages over traditional MS to improve ^13^C-metabolic flux analysis.

## Graphical Abstract

**Graphical Abstract:**
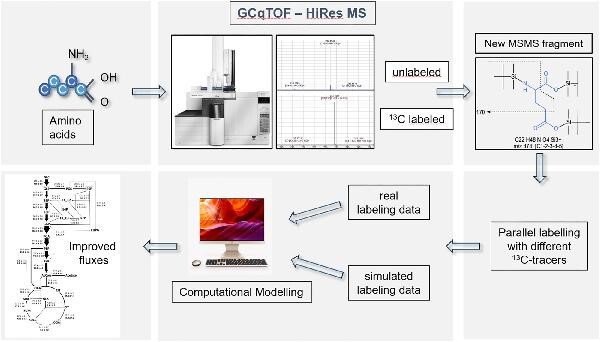
Schematic representation of an analytical workflow, which allowed combination of high-resolution mass spectrometry with ^13^C-labelled biomass to provide positional information in tandem MS amino acid fragments, which led to improved ^13^C-MFA.

## Introduction

Metabolic fluxes reflect the integrated output of genome, transcriptome, proteome, and regulatory interactions, and as such provide a quantitative measure of *in vivo* metabolic phenotypes (Stephanopoulos, 1999; Wiechert, 2001; Sauer, 2006). Over the past 25 years, ^13^C-metabolic flux analysis (^13^C-MFA) has become a key technique for metabolic flux quantification in microbial, plant, and mammalian systems, with a wide range of applications in biotechnology, metabolic engineering, and biomedical sciences (Stephanopoulos, 1999; Schwender, 2008; Allen et al., 2009; Boghigian et al., 2010; Reed et al., 2010; Toya & Shimizu, 2013; Badur & Metallo, 2017; Metallo & Deberardinis, 2017). In ^13^C-MFA, metabolic fluxes are calculated from isotope labeling measurements and extracellular exchange rates using a model-based least-squares regression approach, which identifies an optimal flux distribution that maximizes the fit between the measured experimental data and the predicted model data (Antoniewicz et al., 2006). Classically, the isotopic labeling, introduced by a ^13^C-labeled substrate (the tracer), is measured by mass spectrometry (MS) (Klapa et al., 2003), nuclear magnetic resonance spectroscopy (Szyperski, 1995), and, only recently, by tandem MS (Antoniewicz, 2013). Since the first application of tandem MS data for metabolic flux studies by Jeffrey at al. (2002), several studies have demonstrated the advantage of gas chromatography–tandem mass spectrometry with electron ionization (GC–EI–MS/MS) measured labeling data for ^13^C-MFA application (Choi & Antoniewicz, 2011; Choi et al., 2012; Okahashi et al., 2016; Wang et al., 2021). In GC–EI–MS/MS, the presence of a second fragmentation step yields additional labeling data and positional labeling information, which has shown to significantly improve flux resolution and precision (Antoniewicz, 2013). Despite these demonstrated benefits, so far GC–EI–MS/MS data have only been applied in a few ^13^C-MFA application studies (Okahashi et al., 2016; Wang et al., 2021). Although recent advances have been made in the development of computational approaches to simulate and apply tandem MS data for ^13^C-MFA (Tepper and Shlomi, 2015; Choi & Antoniewicz, 2019), the lack of comprehensive libraries of informative precursor–product ion pairs for accurate ^13^C analysis still hinders the broad application of the tandem MS technique (Antoniewicz, 2013). Apart from studies on GC–EI–MS/MS fragments of single proteinogenic amino acids (Jeffrey et al., 2002: glutamate; Choi et al., 2012: aspartate), only one study investigated GC–EI–MS/MS precursor–product ion pairs of different proteinogenic amino acids with eight fragments being confirmed for ^13^C–MFA (Okahashi et al., 2016).

The aim of this study has therefore been to compile a comprehensive library of GC–EI–MS/MS fragments of 13 amino acids obtained from acid protein hydrolysate. By establishing an analytical workflow, which combines high-mass-resolution gas chromatography–quadrupole time-of-flight mass spectrometry (GC–EI–QTOF) and fully ^13^C-labeled biomass to identify and structurally elucidate tandem MS amino acid fragments, we were able to compile a comprehensive library of 129 precursor–product ion pairs of 13 proteinogenic amino acids with 30 fragments being confirmed for ^13^C-MFA. The suitability of the developed method is demonstrated by a proof-of-concept study employing 14 novel GC–EI–MS/MS fragments for metabolic flux quantification. This case study confirms the importance of the novel tandem MS data for high-resolution ^13^C-MFA, thus indicating that the compiled library should make an important contribution to the routine application of tandem MS data for biomass-based ^13^C-MFA.

## Material and Methods

### Chemicals

Chemicals were purchased from ROTH and Sigma–Aldrich. Glucose tracers, 1-^13^C and U-^13^C glucose, were purchased from Campro Scientific GmbH. The sterile M9 growth medium was prepared as described in Wilms et al. (2001; see Supplementary Data A5 for details).

### Strains and Growth Conditions

The *Escherichia coli* Dh5α strain (Thermo Fisher Scientific, MA, USA) was used for this study. The pre-culture, aerobically grown from a single colony on a M9 medium (Wilms et al., 2001; Supplementary Data A5) agar plate, was cultured overnight in M9 medium (30 mL) in a 100-mL Erlenmeyer flask (Sigma–Aldrich, St. Louis, MO, USA) at 37°C with constant shaking at 140 rpm in a WiseCube incubator (Witeg, Wertheim, Germany). The pre-culture was inoculated to the main culture containing 11 mmol/L glucose. For the determination of amino acid-derived carbons in the fragment ions (see Supplementary Data A1), U^13^-C glucose was employed as the sole carbon source. For the metabolic flux experiment, a mixture of 20% U-^13^C glucose and 80% 1-^13^C glucose was employed. The cells were inoculated at optical density measured at 600 nm (OD_600_) ∼ 0.03 and cultured aerobically in M9 medium (30 mL) at 37°C in a 100-mL Erlenmeyer flask.

### Analytical Methods

Samples were collected during mid-exponential growth phase (OD_600_ ∼ 1) and centrifuged in 50-mL Greiner tubes (Sarstedt, Nümbrecht, Germany) at 10°C and 6600 × *g* for 10 min. After centrifugation, the resulting supernatant and biomass pellet were stored at −80°C until further analysis.

Glucose uptake, acetate excretion, and the specific growth rate were determined in cultures grown on M9 medium supplemented with unlabeled ([^12^C]) glucose. Supernatant was collected at different time-points along the growth phase.

The glucose concentration in the supernatant was determined by gas chromatography-mass spectrometry with electron ionization (GC-EI-MS) (Lisec et al., 2006). For this, the supernatant was diluted 10-fold with ddH_2_O and 10 µL was dried in a vacuum concentrator (RVC2-33CD; CHRIST, Osterode, Germany) for 15 min. The dried samples were derivatized with methoxamine (20 mg/mL solved in pyridine; Sigma–Aldrich, St. Louis, MO, USA) for 30 min at 45°C. Instead of *N*-Methyl-*N*-(trimethylsilyl)trifluoroacetamide (MSTFA), 45 µL Bis(trimethylsilyl)trifluoroacetamide (BSTFA; Macherey-Nagel, Düren, Germany) was added and the samples were mixed for 2 h. The derivatization was done and scheduled by an MPS-MultiPurpose autosampler (Gerstel, Mühlheim, Germany). Absolute quantification of glucose was measured using GC-EI-QTOFMS (GC: 7890B; MS: 7200 Accurate-Mass Q-TOF MS system, Agilent, Santa Clara, CA, USA). The GC system was equipped with an HP-5MS column (Ultra Inert 30 m, 0.25 mm, 0.25 µm, Agilent, Santa Clara, USA). The helium flow was set to 1 mL/min. The temperature gradient was set to 60°C for 1 min, followed by a linear ramp of 10°C/min to 320°C and holding at this temperature for 3 min. During the run, the liner, transfer line, source, and quadrupole were set to 250°C, 290°C, 230°C, and 150°C, respectively. The ionization was performed with electron impact (EI) ionization and recorded with 6.5 spectra/s. The raw data were processed by the MassHunter Quantitative Analysis software for QTOF (version B.08.00; Agilent, Santa Clara, USA).

Acetate was quantified from the supernatant by high-performance liquid chromatography (HPLC) analysis. The separation was performed using an Ultimate 3000 HPLC (Dionex, Sunnyvale, USA) equipped with an Acclaim capillary column (120-C18, 3 µm, 120 Å, 4.6 × 100 mm, Thermo Fischer Scientific, MA, USA). The gradient program was set with an isocratic flow rate of 0.7 mL/min (15% Acetonitrile and 85% water). The ultraviolet-visible (UV-VIS) detector was set at 210 nm with a collection rate of 2 Hz. Eighty microliters was injected and the column temperature was set to 30°C. A standard curve was used to quantify the respective acetate concentration in the samples.

For the determination of *E. coli* cell dry weight (CDW), OD_600_ was measured using a photometer (GeneQuant 1300; Healthcare Bio-Science AB, Uppsala, Sweden). Specific amounts of the culture were centrifuged for 10 min at 16 900×*g*, lyophilized (Alpha 2-4 LDplus; CHRIST, Osterode, Germany) for 24 h and the CDW calculated by correlation of CDW and OD. Unless described differently, all cultures/experiments were repeated in quadruplicates.

For the GC-EI-MS and GC-EI-MS/MS analysis of proteinogenic amino acids, proteins were extracted from the biomass pellet by homogenizing the pellet with 1 mL protein extraction buffer (0.01 M Na_3_PO_4_; pH 7.5; 0.5 M NaCl) on ice. The homogenate was transferred into a 2 mL Eppendorf flask and 1 mL hexane/diethyl ether (1:1) was added. After vortexing and incubation on ice for 30 min, the raw extract was fractionated into the lipid and protein phases by centrifugation (10 min; 4°C; 16 900 × *g*). The protein fraction was purified by adding 1 mL hexane/diethyl ether (1:1) and by centrifugation (10 min; 4°C; 16 900 × *g*). After discarding the supernatant, the purified protein extract was stored at −80°C until protein hydrolysis.

The proteins were hydrolyzed by adding 10% trichloroacetic acid, incubating for 30 min on ice, and further centrifugation for 10 min at 4°C and 16 900 × *g*. After discarding the supernatant, the pellet was washed three times in 1 mL ethanol/diethylether (1:1) and centrifuged again for 10 min. Each pellet was dried by N_2_ gassing (0.5 L/min; Barkey vapostat; Biosystems, Viersen, Germany). After addition of 1 mL 6 M HCl, the pellet was incubated at room temperature for 1 h. The sample was then shaken at 600 rpm for 16 h at 99°C in a ThermoMixer (Eppendorf, Hamburg, Germany) and stored at −80°C until further analysis.

### GC-EI-MS and GC-EI-MS/MS Analysis of Amino Acid Derivatives

Of the 20 naturally occurring amino acids, 7 amino acids varied in their stability due to various reasons. For example, post-hydrolysis, asparagine and glutamine are converted into aspartate and glutamate, while cysteine and methionine are partially oxidized into cysteic acid and methionine sulfoxide or methionine sulfone, respectively, and tryptophan is completely decomposed (Peace & Gilani, 2005). In addition, treatment with silylation reagents converts arginine to ornithine (Leimer et al., 1977). Accurate and reliable measurements of histidine were also difficult due to its low abundance and thus making the precursor–product ion pair transitions difficult to measure. Remaining 13 amino acids were stable and reliably measured after acid hydrolysis of the protein extracts from the samples. Thus, for derivatization, the proteinogenic amino acids were dried (60°C, N_2_ gassing: 0.5 L/min; Barkey vapostat; Biosystems, Viersen, Germany) in injection vials (Phenomenex, Torrance, USA) and sealed with magnetic caps (Silicon/PTFE, Klaus Trott, Kriftel, Germany). The dried samples were dissolved in 30 μL of acetonitrile (Carl Roth, Karlsruhe, Germany) and derivatized by adding 40 μL of *N*-methyl-*N*-(*tert*-butyldimethylsilyl)-trifluoroacetamide (MTBSTFA; Sigma–Aldrich, St. Louis, MO, USA) for 30 min at 70°C. An aliquot of the supernatant was subjected to GC-EI-MS and GC-EI-MS/MS analysis.

For the identification and structural elucidation of tandem MS amino acid fragments, an Agilent GC-EI-QTOF was used. The system consisted of an Agilent 7890B gas chromatograph equipped with an HP capillary GC Column (HP-5MS Ultra Inert 30 m, 0.25 mm, 0.25 µm; Agilent, Santa Clara, USA), connected to an Agilent 7200 Accurate-Mass Q-TOF–MS with a mass accuracy of <2 ppm in 4 GHz mode. Optimal analysis parameters were as follows: The injector and liner temperatures were set to 250°C. The temperature of the column was started at 60°C (2 min), increased to 280°C at 20°C/min, and maintained for 5 min. GC injection of 1.0 μL aliquots of sample solution was performed. Helium (Air Liquide, Paris, France) was used as the carrier gas at a constant flow rate of 1 mL/min. The ion source temperature was kept at 230°C and the quadrupoles at 150°C. Ionization was performed with EI ionization. The electron energy was set to 70 and 30 eV, respectively. For 70 eV, the filament current was set to a signal strength of 1 000 000 counts, and for 30 eV, it was set to maximum signal strength. For tandem MS/MS analysis, Argon (Air Liquide, Paris, France) was employed for collision induced dissociation at a constant flow rate of 1.5 mL/min. The collision energy was individually optimized between 2 and 20 eV for each product fragment for a high signal intensity and the avoidance of other fragments in the range of the natural isotope distribution of the specific ion. For qualitative analysis, the detector was operated in 4 GHz range mode, for quantitative analysis the 2 GHz range mode was employed. Product spectra were recorded in scan mode (6.5 spectra/sec) and the precursor ions [M-15]^+^, [M-57]^+^, [M-85]^+^, [M-159]^+^, and [f302]^+^ with their typical loss were selected for each amino acid (Chaves Das Neves & Vasconcelos, 1987; Supplementary Data A2, Supplementary Fig. S1).

For ^13^C metabolic flux analysis, the single- and tandem-MS analyses were performed using an Agilent GC-EI-MS/MS. The system consisted of an Agilent 7890 GC equipped with a HP-5 capillary column (30 m; 0,25 mm, 0,25 μm; Agilent, Santa Clara, USA), interfaced with a triple quadrupole tandem mass spectrometer (Agilent 7000B) operating under EI ionization at 70 eV. GC parameters were as described above. For GC-EI-MS analysis, mass isotopomer distributions (MIDs) were recorded in selected ion monitoring mode with 20 ms dwell time. For GC-EI-MS/MS analysis, argon gas was used as the collision gas and the product spectra were recorded in MRM mode with 20 ms dwell time.

For GC-EI-MS and GC-EI-MS/MS analysis, MIDs were obtained by integration using the MassHunter Quantitative Analysis software (version B08.00; Agilent Technologies, CA). The tandem mass isotopomer distribution (TMID) as well as MID of the product fragments was calculated as described in Choi & Antoniewicz, (2011). Measured mass isotopomer distributions were corrected for natural isotope abundances using iMS2Flux (Poskar et al., 2012, version v.7.2.1). The ^13^C-labeling of glucose tracers was experimentally determined by GC-EI-MS as described in Long & Antoniewicz (2019) (see Supplementary Data A5 for details).

### Labeling Data Validation

To validate the mass isotopomer distributions of the proteinogenic amino acids measured by GC-EI-MS and GC-EI-MS/MS analysis, the quality of the labeling data was quantified based on measurement accuracy and precision as detailed in Antoniewicz et al. (2007a). To quantify the measurement accuracy of the detected candidate fragment ions, the theoretical TMID for each precursor–product ion pairs was calculated as described in Choi et al. (2012) and compared to the measured TMID. For GC-EI-MS and GC-EI-MS/MS data, fragments with an accuracy <0.4 mol% and precision <0.4 mol% were considered accurate and applied for ^13^C-MFA.

To compare the information content resulting from GC-EI-MS versus GC-EI-MS/MS-based isotopomer analysis, the number of independent constraints (NICs) on the isotopomer distribution of the experimentally identified GC-EI-MS and GC-EI-MS/MS fragments was calculated. For an amino acid with *n* carbons, 2*^n^* independent constraints are required to resolve the abundance of all 2*^n^* isotopomers of the amino acid (Dauner & Sauer 2000). As described previously (Dauner & Sauer 2000; Choi et al., 2012), the number of independent constraints for a selected set of *(tandem)* MS fragments is equal to the rank of the matrix *N*, which contains the theoretical *(T)*MIDs for all the selected *(tandem)* MS fragments:


(1) \begin{equation*}{\mathrm{NIC}} = rank\left( N \right).\end{equation*}

Using equation (1), the number of independent constraints on the isotopomer distribution of the experimentally identified GC-EI-MS and GC-EI-MS/MS fragments for each of the studied proteinogenic amino acids was calculated as detailed in Supplementary Data A4.

In addition, GC-EI-MS versus GC-EI-MS/MS-based isotopomer analysis was compared with respect to the number of independent measurements (NIMs). For MS analysis, the NIMs for a given MS fragment of *n* carbon atoms corresponds to n. For tandem MS analysis, the NIMs is (*n* – *m* + 1) (*m* + 1) – 1, whereby *n* corresponds to the carbon atoms of the precursor ion and *m* to the carbon atoms of the product ion (Choi & Antoniewicz, 2011).

### ^13^C–Metabolic Flux Analysis of *E. Coli*

#### Metabolic Model

A detailed metabolic model of *E. coli* primary metabolism was constructed by reviewing the respective literature and online databases (e.g., Neidhardt et al. 1990; Karp et al., 2018). The model comprises glycolysis, the tricarboxylic acid (TCA) cycle, the pentose phosphate pathway, anaplerotic reactions, the glyoxylate shunt, the Entner–Doudoroff pathway, and amino acid metabolism (Supplementary Data A5). To account for the exchange of atmospheric (unlabeled) and intracellular (labeled) CO_2_, a CO_2_ exchange reaction was included into the model. In addition, G-value parameters for each measured amino acid were integrated to correct for the dilution of labeling resulting from unlabeled proteinogenic amino acids of the inoculum (Antoniewicz et al., 2007c). The biosynthesis of *E. coli* biomass, modeled as lumped biomass formation reaction, was calculated from precursor requirements as described by Neidhardt et al. (1990, pp. 135–136).

#### ^13^C–Metabolic Flux Analysis

Metabolic fluxes were computed using the INCA software (Young, 2014), a Matlab–based flux estimation tool using the elementary metabolite unit (EMU) framework (Antoniewicz et al., 2007b; Young et al., 2008). The experimentally measured extracellular rates and mass isotopomer distributions applied for ^13^C-MFA are given in the Supplementary Data A5. The standard deviation of the applied labeling data measured by GC-EI-MS and GC-EI-MS/MS was assumed to be 0.004 (Antoniewicz et al., 2007a, Wasylenko & Stephanopoulos 2013). In all cases, flux estimation was repeated at least 100 times with random initial start values to find a global optimum. To assess the goodness-of-fit, a  *χ*^2^-test was performed for each optimization. Accurate 95% confidence intervals were calculated for all estimated fluxes as described by Antoniewicz et al. (2006).

## Results and Discussion

### Identification and Structural Elucidation of Tandem MS Amino Acid Fragments

Tandem MS analysis supports the identification of positional information on carbon atoms in MS fragments, which is a prerequisite to improve ^13^C-based flux estimation. To compile a comprehensive library of all possible precursor–product ion pairs, product ion scans for 13 proteinogenic amino acids required for the ^13^C-MFA of the central carbon metabolism in microbes (Antoniewicz et al., 2007a; Okahashi et al., 2014; Long & Antoniewicz, 2019) were acquired. In detail, unlabeled and fully ^13^C-labeled proteinogenic amino acids extracted from *E. coli* cultured in a medium containing ^12^C and U-^13^C glucose, respectively, were derivatized with *tert*butyldimethylsilyl (TBDMS) and subjected to high-resolution GC-EI-QTOF analysis. Candidate MS/MS fragment ions were selected by screening the product ion spectra of the precursor ions [M-15]^+^, [M-57]^+^, [M-85]^+^, [M-159]^+^, and [f302]^+^. Each product ion spectrum was analyzed manually to extract the accurate mass of the most abundant fragment ions. For each of the detected candidate fragments, the sum formula of the fragment ion was identified based on ppm-value analysis (see Supplementary Data A1 for details). The number of amino acid-derived carbons in a candidate fragment ion was determined by comparing the *m/z* values of the unlabeled versus fully ^13^C-labeled fragment of identical sum formula as the *m/z* value difference corresponds to the number of labeled ^13^C atoms in the amino acid carbon backbone (Supplementary Fig. S2).

**Fig. 1.**
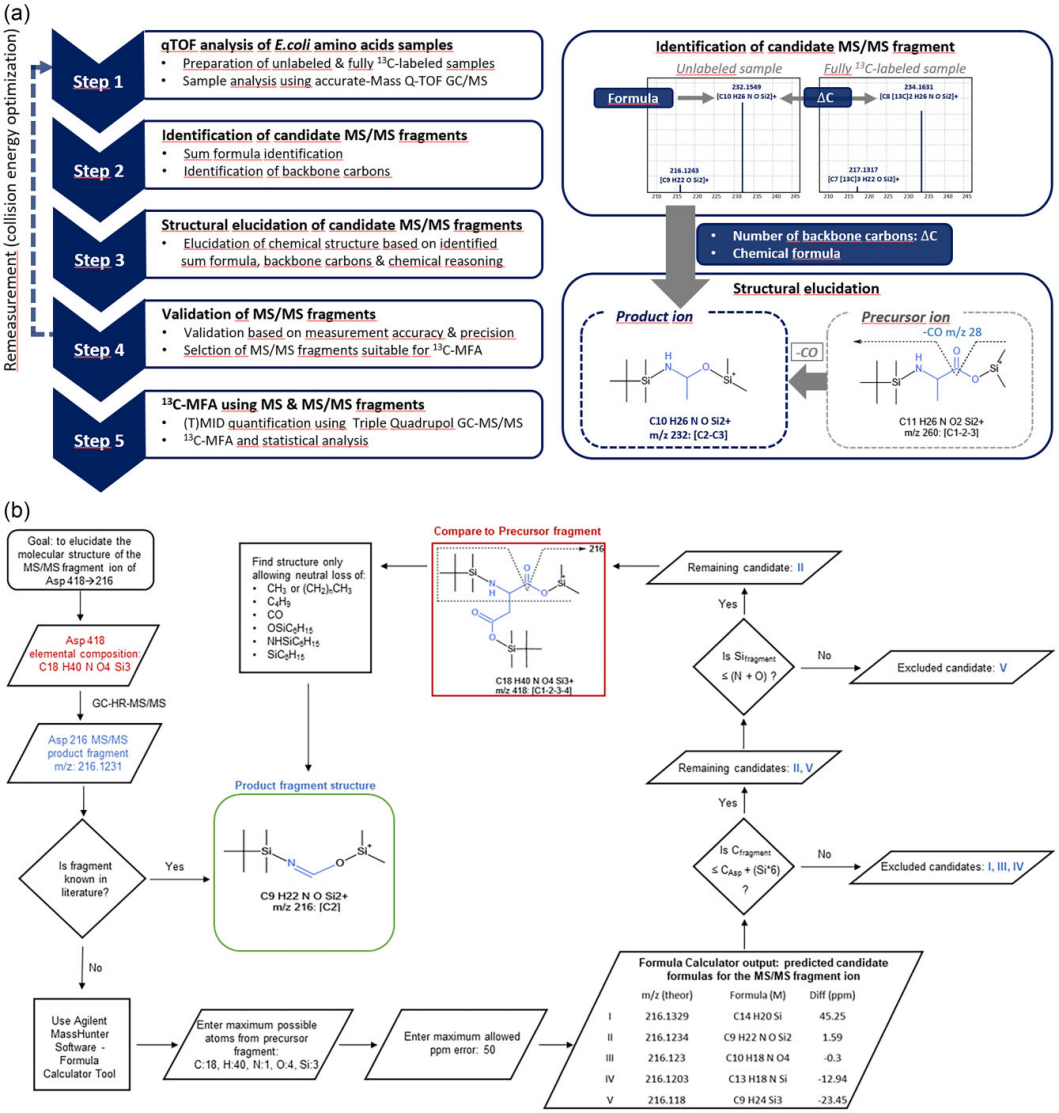
(a) Workflow of the high mass accuracy MS procedure for tandem MS fragment identification. (b) A workflow process for determining the molecular structure of each candidate MS/MS fragment ion (e.g., TBDMS-derivatized aspartate).

The molecular structure of each candidate MS/MS fragment ion was postulated using the information of (1) the candidate sum formulas derived from precise mass using high mass accuracy MS, (2) the amino acid–derived carbon atoms by comparing the ^13^C/^12^Cdifference of the amino acid backbone, (3) maximum candidate elemental composition compared with the precursor fragment, and (4) the limitation of structure possibilities caused by the number of C atoms and the dependence of the binding of Si atoms to the functional groups (rules in Supplementary Data A1, Fig. 1a and b). Candidates with non-conclusive, ambiguous data were discarded. For the resulting set of candidate fragment ions, the process of structural elucidation as well as the resulting chemical structure of each fragment ion (including the positional origin of the carbon atoms) is detailed in the Supplementary Data A2.

### Fragment Ion Validation

To validate the novel high–mass accuracy MS procedure for tandem MS fragment identification (Fig. 1a), the quality of the detected candidate fragment ions was quantified based on the measurement accuracy and precision of each fragment as described in Section “Labeling Data Validation”. Fragments for which the measurement accuracy was >5 mol% were considered inaccurate and were discarded from further analysis. To ensure comprehensiveness by maximizing the measurement accuracy of each fragment ion, the tandem MS analysis was performed on two different mass spectrometers GC-EI-QTOF, GC-EI-MS/MS and the collision energy was optimized for each product fragment. However, not all fragments of the amino acids could be validated, because the dwell time of all transitions of them would be too short. For example, the precursor–product ion pair of phenylalanine *m/z* 378 > *m/z* 350 needs 81 transitions to resolve all possibilities.

### Library of Precursor–Product Ion Pairs

The high mass accuracy MS procedure for tandem MS fragment identification described above resulted into the identification of 129 validated precursor–product ion pairs of 13 proteinogenic amino acids. In total, 30 of these fragments were accepted for ^13^C-MFA (i.e., measurement accuracy <0.4 mol%; see Table 1). The complete library of precursor–product ion pairs is given in Supplementary Data A3.

**Table 1.** Tandem MS Fragments Identified by the High–Mass Accuracy MS Procedure, Which Were Accepted for ^13^C–MFA

	**Precursor ion**			**Product ion**				**References**
**Amino acid**	*m/z*	Fragment ion	Carbon atoms	*m/z*	Carbon atoms	Measurement accuracy^a^ Δ_max_ [mol%]	Measurement precision^b^ (*n* = x) Std Dev_max_ [%]	Related work
Ala	260	[M-57]+	[1–2-3]	232	[2–3]	0.18	0.09	
Ala	260	[M-57]+	[1–2-3]	158	[2–3]	0.08	0.09	
Ala	260	[M-57]+	[1–2-3]	103	[1]	0.26	0.12	Okahashi et al. (2016)
Asp	418	[M-57]+	[1–2-3–4]	244	[1–2]	0.12	0.09	Okahashi et al. (2016); Choi et al. (2012)
Asp	418	[M-57]+	[1–2-3–4]	216	[2]	0.31	0.10	Choi et al. (2012)^c^
Asp	418	[M-57]+	[1–2-3–4]	103	[1]	0.39	0.22	Choi et al. (2012)
Asp	390	[M-85]+	[2–3-4]	346	[2–3]	0.39	0.34	Okahashi et al. (2016); Choi et al. (2012)
Asp	302	[f302]+	[1–2]	218	[2]	0.32	0.08	Okahashi et al. (2016)
Glu	404	[M-85]+	[2–3-4–5]	244	[2–3-4]	0.13	0.22	Okahashi et al. (2016)
Glu	404	[M-85]+	[2–3-4–5]	170	[2–3-4]	0.11	0.27	Okahashi et al. (2016)
Glu	330	[M-159]+	[2–3-4–5]	170	[2–3-4]	0.21	0.04	Okahashi et al. (2016)
Gly	246	[M-57]+	[1–2]	218	[2]	0.30	0.17	
Gly	246	[M-57]+	[1–2]	103	[1]	0.14	0.07	Okahashi et al. (2016)
Ile	302	[M-57]^+^	[1–2-3–4-5–6]	274	[2–3-4–5-6]	0.36	0.27	
Ile	302	[M-57]+	[1–2-3–4-5–6]	200	[2–3-4–5-6]	0.27	0.14	
Leu	302	[M-57]+	[1–2-3–4-5–6]	274	[2–3-4–5-6]	0.22	0.05	
Lys	302	[f302]+	[1–2]	218	[2]	0.25	0.04	Okahashi et al. (2016)
Phe	302	[f302]+	[1–2]	218	[2]	0.37	0.18	Okahashi et al. (2016)
Pro	286	[M-57]+	[1–2-3–4-5]	258	[2–3-4–5]	0.26	0.11	
Ser	390	[M-57]+	[1–2-3]	362	[1–2]	0.17	0.12	
Ser	302	[f302]+	[1–2]	218	[2]	0.12	0.35	Okahashi et al. (2016)
Thr	404	[M-57]+	[1–2-3–4]	376	[2–3-4]	0.31	0.11	
Thr	302	[M-159]+	[2–3-4]	218	[2]	0.33	0.10	
Thr	302	[M-159]+	[2–3-4]	142	[2]	0.16	0.18	
Thr	302	[f302]+	[1–2]	218	[2]	0.33	0.10	
Thr	302	[f302]+	[1–2]	142	[2]	0.16	0.18	
Tyr	302	[f302]+	[1–2]	142	[2]	0.25	0.18	
Val	288	[M-57]+	[1–2-3–4-5]	260	[2–3-4–5]	0.27	0.09	
Val	288	[M-57]+	[1–2-3–4-5]	186	[2–3-4–5]	0.26	0.33	
Val	302	[f302]+	[1–2]	218	[2]	0.38	0.34	Okahashi et al. (2016)

^a^Maximum difference between measured and predicted TMIDs.

^b^Maximum standard deviation of all replicates.

^c^Choi et al. (2012): unclear if atom C3 is present in a fragment.

To confirm the identified tandem MS fragments, we compared our data with previously published fragments, which were analyzed via GC–EI–MS/MS and the amino acid–derived carbons identified by the analyses of ^13^C positionally labeled authentic standards (Choi et al., 2012; Okahashi et al., 2016). All of these fully resolved published fragments were identified using our high–mass accuracy MS procedure, indicating that our library of precursor–product ion pairs provides a comprehensive list of tandem MS fragments identifiable by current state-of-the art mass spectrometry techniques. In addition, all of the postulated metabolite fragmentation patterns were confirmed by published data, indicating the validity of our approach. Besides resolving 102 novel tandem MS fragments (see Supplementary Data A3), we were also able to resolve the fragmentation pattern of four previously unresolved published fragments (see Supplementary Data A2). For example, the amino acid-derived carbon backbone for the product fragment *m/z* 216 of the precursor fragment *m/z* 418 of aspartate, with hitherto-not-confirmed origin (Choi et al., 2012), was determined again to be the C2 atom based on the high mass accuracy of sum formula prediction applied in our approach (see Supplementary Data A1 and A2).

The amount of ^13^C-labeling incorporation and the distribution of ^13^C-labeled atoms within key metabolites are crucial for high-resolution ^13^C-MFA (Choi & Antoniewicz, 2011; Antoniewicz, 2013). As shown in Table 2, the information content (i.e., NIC) resulting from tandem MS fragments is equal or higher compared to MS fragments for all proteinogenic amino acids (except for proline, most likely due to its cyclic nature). While the isotopomer constitution of small amino acids like serine and glycine was completely resolved using either MS or tandem MS fragments alone, the complete isotopomer distribution of alanine and aspartate could only be resolved using tandem MS fragments. Although the isotopomer constitution of the remaining amino acids could not be fully resolved, a much higher number of isotopomers were determined for most of the amino acids using tandem MS fragments.

**Table 2.** NICs on the Isotopomer Distribution and NICs by MS and Tandem MS Data, in theory, possible

		Number of independent constraints (NICs)^a^		Number of independent measurements (NIMs)	
Amino acid	Number of positional isotopomers	MS data	MS/MS data	MS data^b^	MS/MS data^c^
Glycine	4	4	4	6	11
Alanine	8	6	8	10	33
Serine	8	8	8	12	26
Aspartate	16	12	16	16	88
Threonine	16	8	12	14	48
Glutamate	32	16	19	20	46
Proline	32	16	12	20	20
Valine	32	16	16	20	38
Isoleucine	64	12	18	24	67
Leucine	64	12	24	24	86
Lysine	64	20	37	24	56

The amino acids tyrosine and phenylalanine were not included in the table, as for these amino acids only tandem MS fragments comprising the [f302]^+^ precursor ion were identified due to the ring structure of the amino acids (see Supplementary Data A3). The calculation was restricted to fragments of quality levels I–III.

^a^Fragments used for NIC calculation are given in Supplementary Data A4.

^b^Fragments used for NIM calculation: [M-15]^+^, [M-57]^+^, [M-85]^+^, [M-159]^+^, and [f302]^+^.

^c^Fragments used for NIM calculation are depicted in Supplementary Data A3.

Another advantage of tandem MS compared to full spectrum MS derives from the increased NIMs (Choi & Antoniewicz, 2011). As depicted in Table 2, the NIMs for tandem MS data is significantly higher for all proteinogenic amino acids, thereby offering more informative data for metabolic flux quantification.

Furthermore, GC-EI-MS/MS-based labeling data analysis provides a means of integrating the MIDs of the parent and daughter ions in addition to the tandem MID of a given fragment. As previously shown (Wang et al., 2021), the combination of TMIDs and MIDs of parent and daughter ions is more powerful than using TMID alone and can further improve the precision of flux estimation.

### Application Example: ^13^C–MFA of Central Carbon Metabolism of *E. coli*: MS Versus MS + MS/MS

**Fig. 2.**
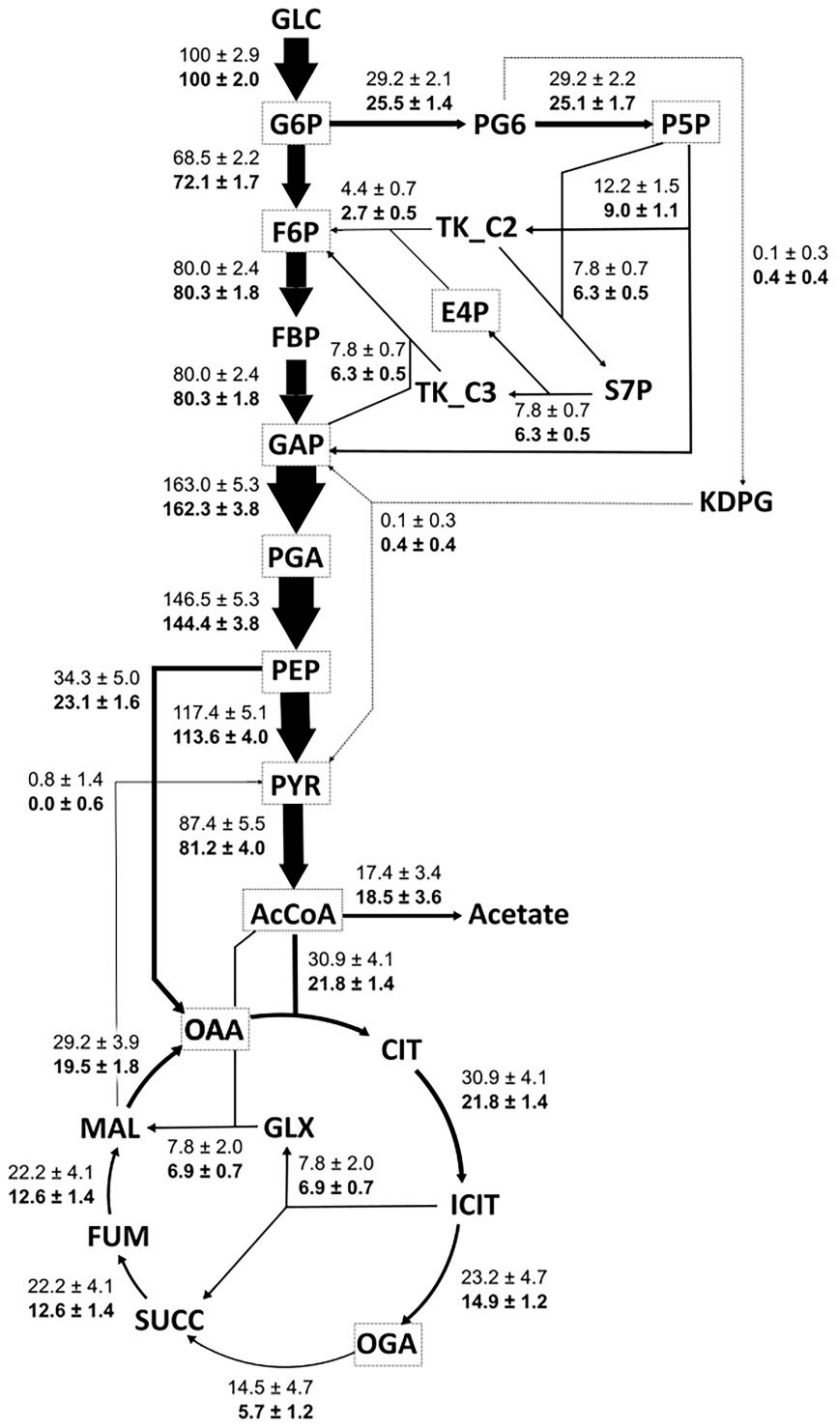
Metabolic flux distributions of *E. coli* grown in aerobic batch culture. Shown are the estimated fluxes using GC-EI-MS and GC-EI-MS/MS data of proteinogenic amino acids, normalized to the glucose uptake rate. The arrow thickness corresponds to the estimated relative flux value. Metabolites highlighted by a dotted line rectangle represent biomass precursors drained for biomass production. Values represent the optimum relative flux value ± standard error. The top values denote the metabolic fluxes determined by GC-EI-MS data and the lower bold values denote the fluxes determined by GC-EI-MS and GC-EI-MS/MS data.

To assess the advantage of tandem MS for ^13^C-MFA, compared to full spectrum MS, metabolic fluxes were determined using (1) MS data and (2) a combination of MS and tandem MS data. In the first case, ^13^C-MFA of the central carbon metabolism of *E. coli* was performed using MID data of the conventional set of amino acid fragments with 88 independent measurements determined by GC-EI-MS. In the second case (using same samples), additional to the GC-EI-MS dataset, 132 independent measurements were determined by GC-EI-MS/MS. Additional MID data for 14 fragments, which were identified by the above-mentioned high mass accuracy tandem MS fragment identification procedure using GC-EI-MS/MS, were applied for flux determination. Assuming a standard deviation of 0.4 mol% for the GC-EI-MS and GC-EI-MS/MS dataset, statistically acceptable fits were obtained for both cases. Figure 2 shows the metabolic flux distributions for both datasets. All data required for ^13^C-MFA as well as the MFA results are given in the Supplementary Data A5. The estimated flux values were similar for each dataset and corresponded well to values reported for *E. coli* grown under similar cultivation conditions (Crown et al., 2015; Okahashi et al., 2016). The comparison of the relative flux values showed a general reduction of the confidence interval due to the additional integration of the GC-EI-MS/MS dataset (Supplementary Data A5—Flux distributions & CI). Significant reduction of the confidence intervals was observed for glycolysis (about 28%), the TCA cycle (up to 67%), different anaplerotic reactions and the glyoxylate shunt (about 65%), but not for the ED pathway and acetate metabolism. In agreement with previous studies (Choi & Antoniewicz 2011; Ruhl et al., 2012; Antoniewicz 2013; Okahashi et al., 2016), these results confirm the significant improvement of flux resolution and precision due to tandem MS data integration.

## Conclusion and Outlook

Tandem MS provides rich information on stable-isotope labeling for metabolic flux quantification, which has shown to significantly improve flux precision and resolution (Choi & Antoniewicz, 2011, Choi et al., 2012; Okahashi et al., 2016). In this paper, we have presented a high mass accuracy MS procedure, which allowed us to compile a library of precursor–product ion pairs of TBDMS-derivatized proteinogenic amino acids. To provide a comprehensive list of tandem MS fragments identifiable by current state-of-the-art mass spectrometry techniques, high-resolution GC–EI–QTOF was applied for fragment identification. Next, high-resolution ^13^C–MFA was performed based on these novel fragments by measuring the stable-isotope labeling via GC–EI–MS/MS. In addition to providing a higher reproducibility of the isotope ratios compared to GC-EI-QTOF, cost-effective GC–EI–MS/MS has the advantage of being more widely distributed, thus supporting the routine application of tandem MS data for ^13^C-MFA.

As shown in this paper, tandem MS fragments provide positional information and additional labeling information resulting from a higher information content and an increased NIM, therefore offering significant advantages over traditional MS. In the future, it would also be beneficial to further determine the relative impact of several MS fragment combinations on MFA to improve our understanding of the biochemical insights and the rationale of using tandem MS analysis for better metabolic flux resolution. Together with the recent algorithmic advances in tandem MS data integration into ^13^C-MFA and the evolving availability of comprehensive libraries of MS/MS fragments (amino acids, organic acids, sugar phosphates, etc.), we anticipate that tandem MS measurements will find widespread usage for high-resolution ^13^C-MFA.

## Supplementary Material

kuad039_Supplemental_Files
