# Supplementary material for: Improved 13C metabolic flux analysis in Escherichia coli metabolism: application of a high-resolution MS (GC–EI–QTOF) for comprehensive assessment of MS/MS fragments
Source: J Ind Microbiol Biotechnol. 2023 Nov 13;50(1):kuad039. doi: 10.1093/jimb/kuad039 (PMC10716738; doi:10.1093/jimb/kuad039)

**Supplementary Fig.1: *Structural elucidation depicting loss of functional groups in the TBDMS-derivatised amino acid***

The molecular structure of a precursor ion was elucidated using the TBDMS-derivatised asparate as an example to depict the loss of functional groups for each product fragment.


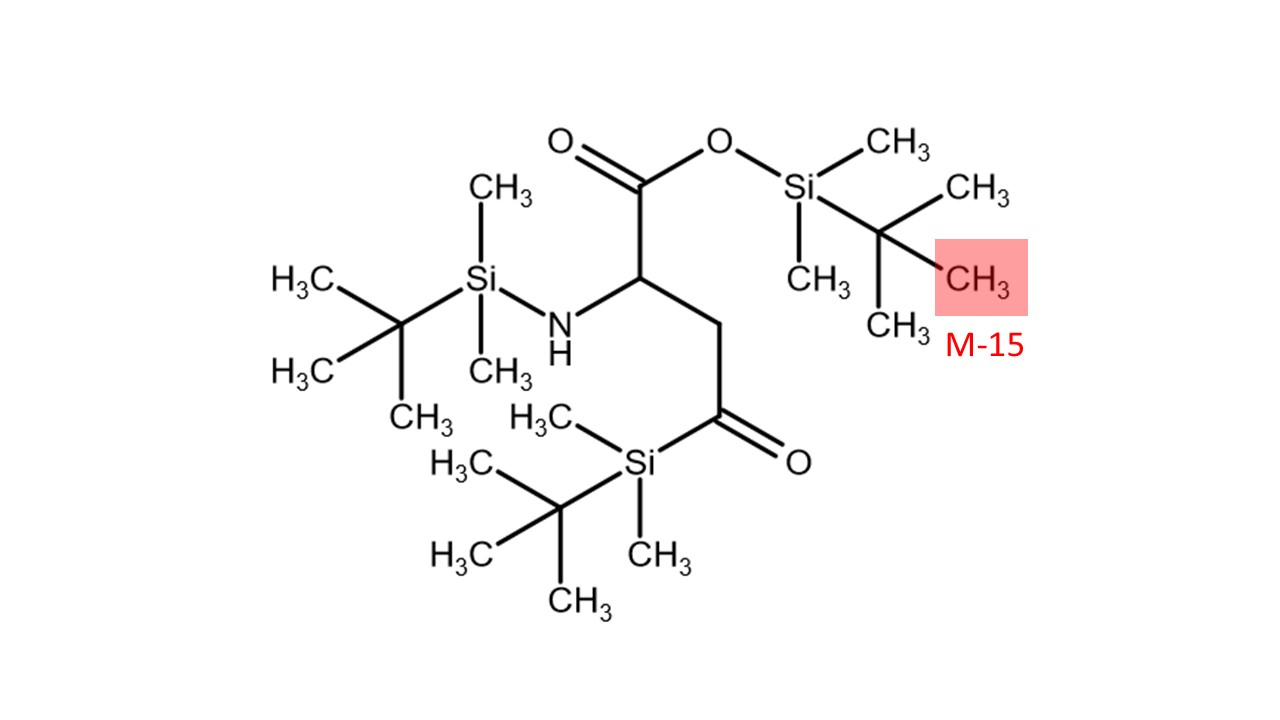

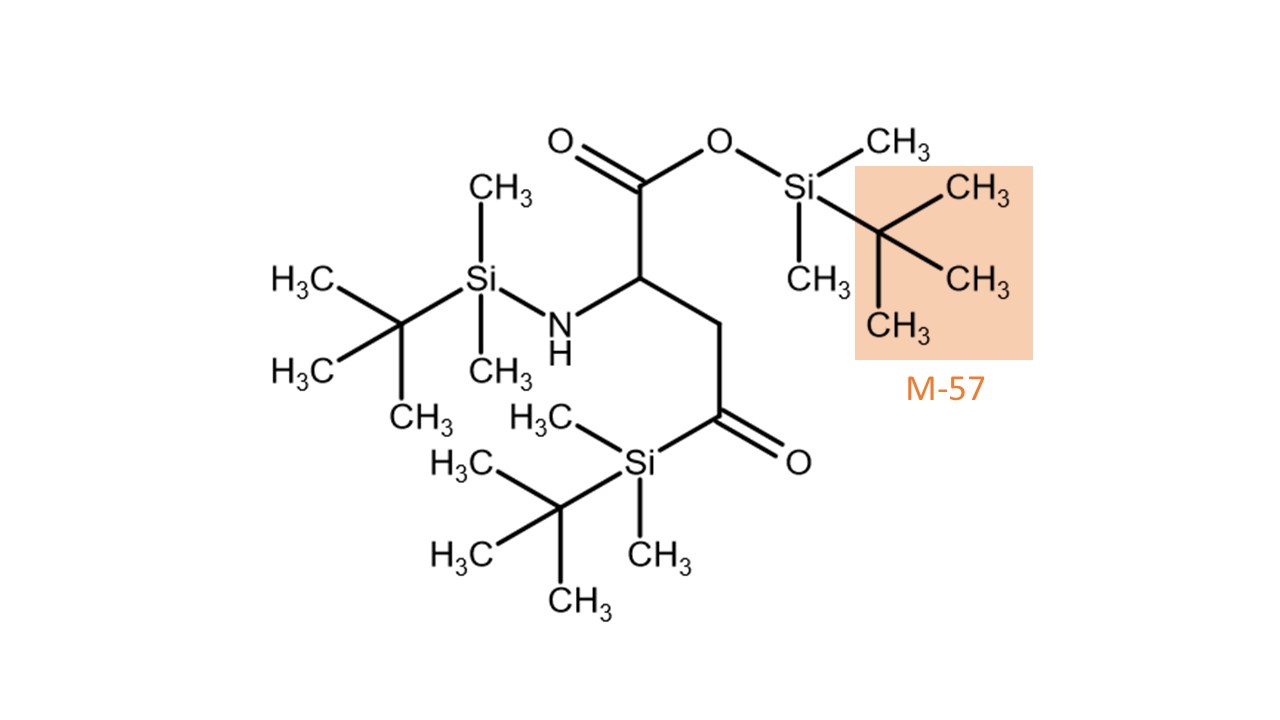

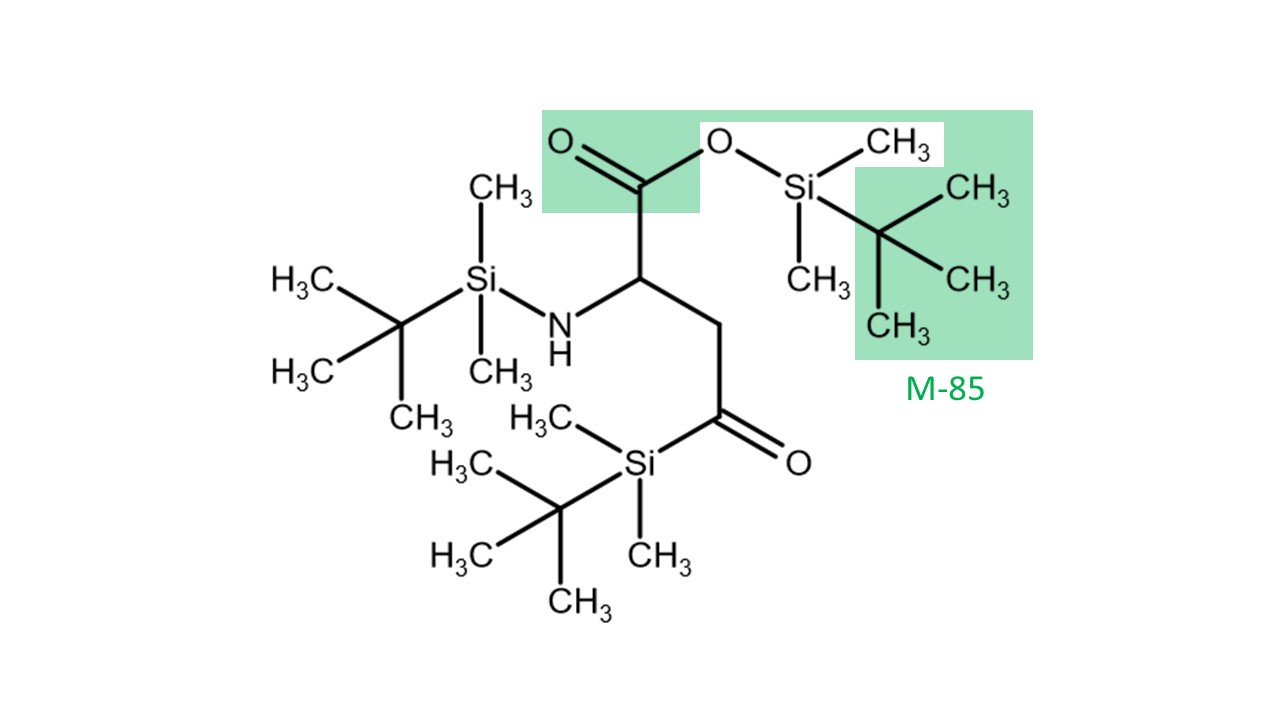

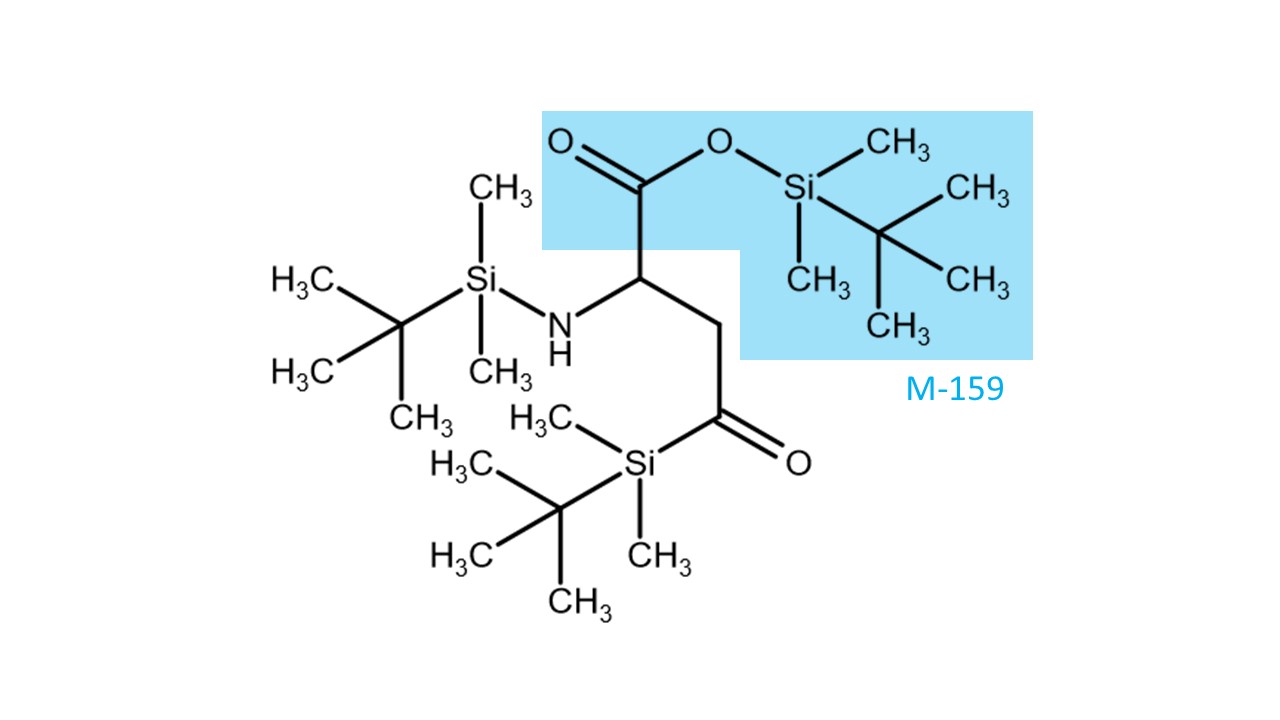


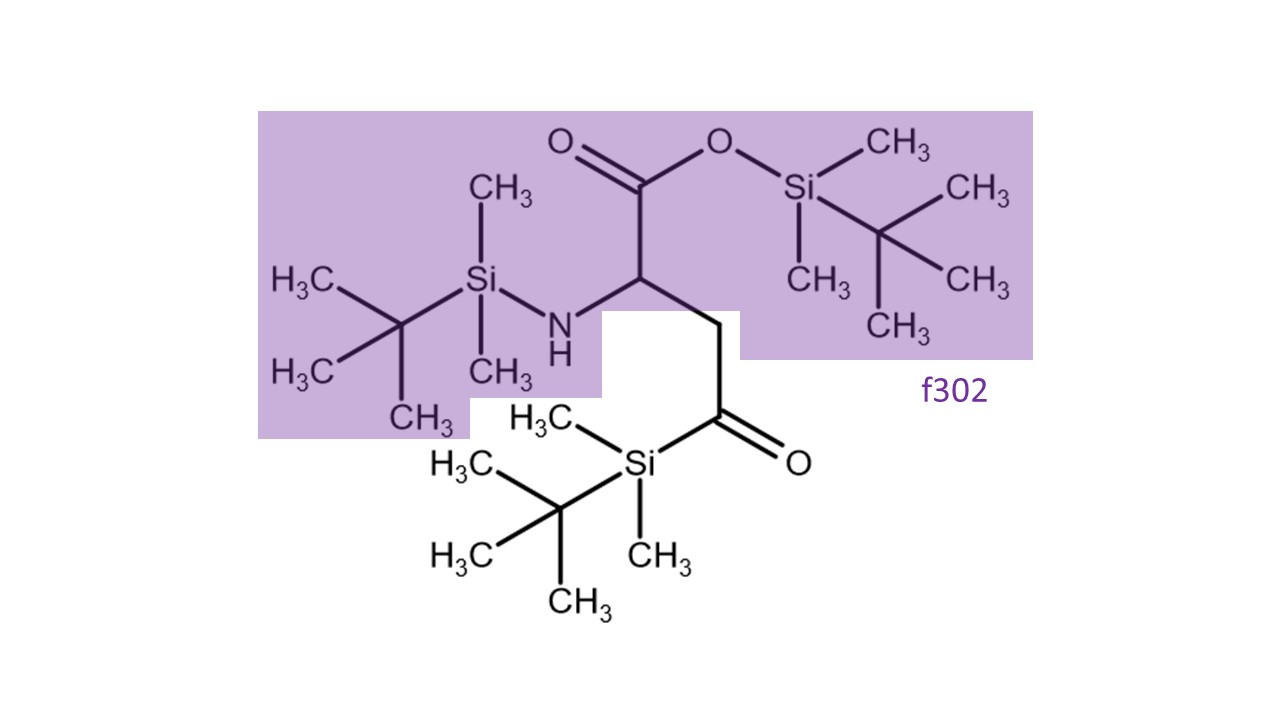

Supplement: kuad039_Supplemental_Files [file kuad039_supplemental_files.zip › Supplementary Figure 1.docx]
