# Supplementary material for: Improved 13C metabolic flux analysis in Escherichia coli metabolism: application of a high-resolution MS (GC–EI–QTOF) for comprehensive assessment of MS/MS fragments
Source: J Ind Microbiol Biotechnol. 2023 Nov 13;50(1):kuad039. doi: 10.1093/jimb/kuad039 (PMC10716738; doi:10.1093/jimb/kuad039)
Supplement: kuad039_Supplemental_Files [file kuad039_supplemental_files.zip › Supplementary Figure 2.docx]

**Identification of candidate MS/MS fragments:**

***Determination of the number of backbone carbons in product ions***

The molecular structure of a candidate product ion was elucidated using the information of the sum formula derived from high-mass accuracy MS, the number of amino acid-derived carbons in the fragment, and biochemical reasoning. To identify the number of backbone carbons, uniformly ^13^C-labeled biomass of *E.coli* grown in [U^13^-C]glucose was analyzed. By feeding cells with fully labeled glucose as the sole carbon source, the resulting amino acids (i.e. their backbone carbons) become uniformly labeled. As the mass of the daughter ion will change according to the number of backbone carbons, the comparison of the m/z value of the unlabeled versus fully ^13^C-labeled fragment allows the determination of amino acid-derived carbons in the fragment as depicted in the example below:
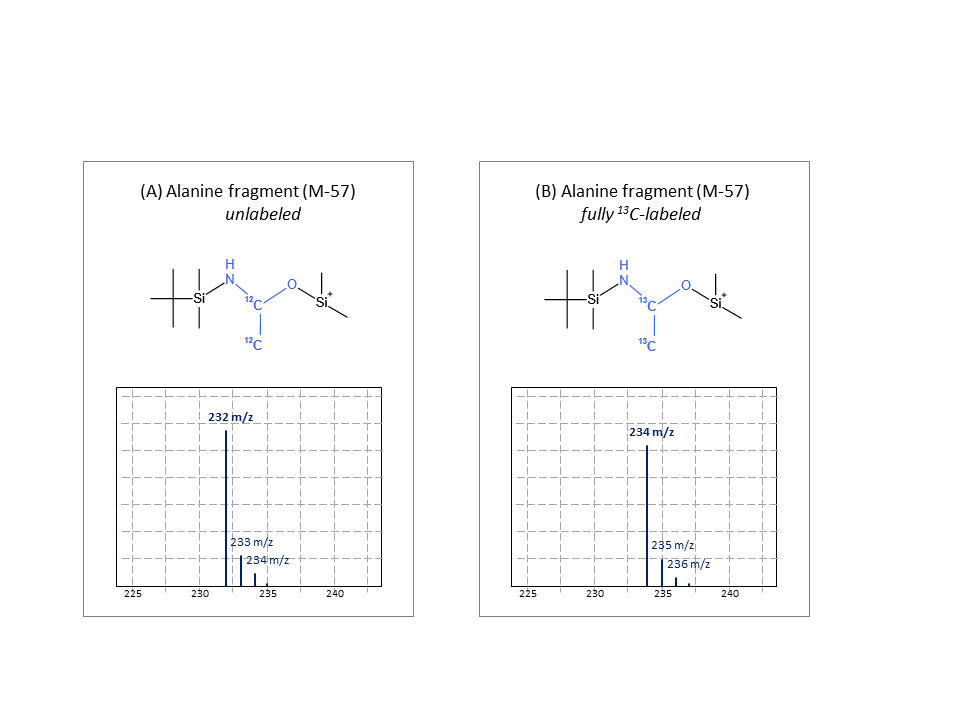


**Supplementary Fig. 2:** Comparison of the m/z value of (A) unlabeled and (B) fully ^13^C-labeled M‑57 fragment of alanine: molecular structures and mass spectra.
