# Supplementary material for: Improved 13C metabolic flux analysis in Escherichia coli metabolism: application of a high-resolution MS (GC–EI–QTOF) for comprehensive assessment of MS/MS fragments
Source: J Ind Microbiol Biotechnol. 2023 Nov 13;50(1):kuad039. doi: 10.1093/jimb/kuad039 (PMC10716738; doi:10.1093/jimb/kuad039)
Supplement: kuad039_Supplemental_Files [file kuad039_supplemental_files.zip › SupplementaryData A2.docx]

| **Amino acid**  [m/z transition] | **Precursor ion**  (structure, sum formula, m/z) | **Product ion**  (structure, sum formula, m/z) | **Neutral loss** | **Confidence level** |
| --- | --- | --- | --- | --- |
|  |  |  |  |  |
| **Glycine**  *[246>218]* | 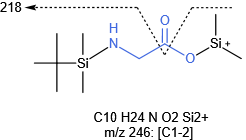 | 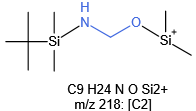 | CO | 2 |
| **Glycine**  *[246>103]* | 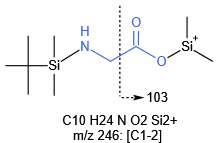 | 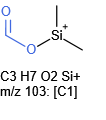 | C7H17NSi | 1  (Okahashi et al., 2016) |
| **Glycine**  *[246>88]* | 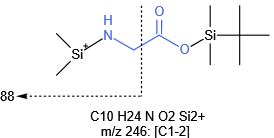 | 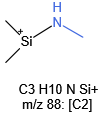 | C7H14O2Si | 2 |
| **Glycine**  *[246>86]* | 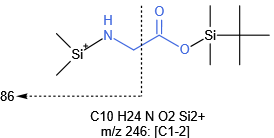 | 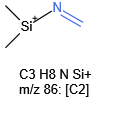 | C7H16O2Si | 2 |

**Supplementary Data A2:** The process of structural elucidation as well as the resulting chemical structure of each fragment ion (including the positional origin of the carbon atoms)

| **Amino acid**  [m/z transition] | **Precursor ion**  (structure, sum formula, m/z) | **Product ion**  (structure, sum formula, m/z) | **Neutral loss** | **Confidence level** |
| --- | --- | --- | --- | --- |
|  |  |  |  |  |
| **Alanine**  *[302>232]* | 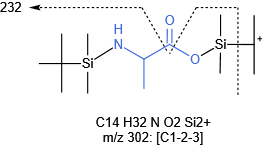 | 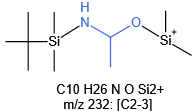 | C4H6O | 2 |
| **Alanine**  *[302>103]* | 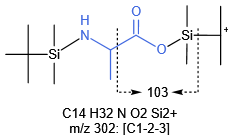 | 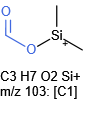 | C11H25NSi | 2 |
| **Alanine**  *[260>232]* | 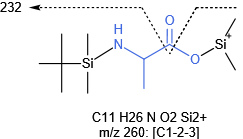 | 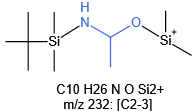 | CO | 2 |
| **Alanine**  *[260>158]* | 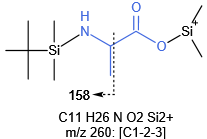 | 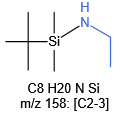 | C3H6O2Si | 2 |
| **Alanine**  *[260>103]* | 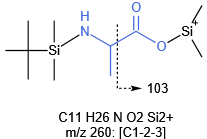 | 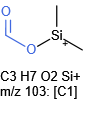 | C8H19NSi | 1  (Okahashi et al., 2016) |

| **Amino acid**  [m/z transition] | **Precursor ion**  (structure, sum formula, m/z) | **Product ion**  (structure, sum formula, m/z) | **Neutral loss** | **Confidence level** |
| --- | --- | --- | --- | --- |
|  |  |  |  |  |
| **Serine**  *[390>362]* | 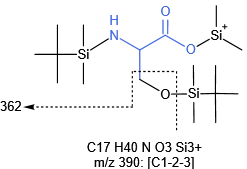 | 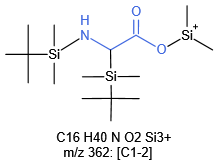 | CO | 2 |
| **Serine**  *[390>288]* | 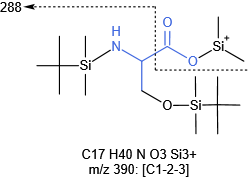 | 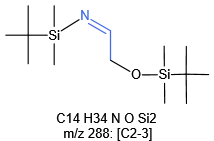 | C3H6O2Si | 2 |
| **Serine**  *[390>230]* | 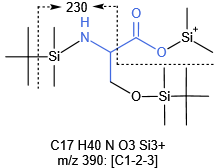 | 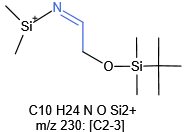 | C7H16O2Si | 2 |
| **Serine**  *[390>142]* | 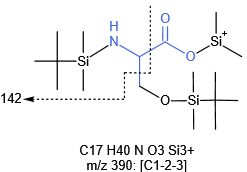 | 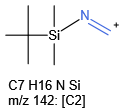 | C10H24O3Si2 | 2  (Okahashi et al., 2016) |

| **Amino acid**  [m/z transition] | **Precursor ion**  (structure, sum formula, m/z) | **Product ion**  (structure, sum formula, m/z) | **Neutral loss** | **Confidence level** |
| --- | --- | --- | --- | --- |
|  |  |  |  |  |
| **Aspartate**  *[418>390]* | 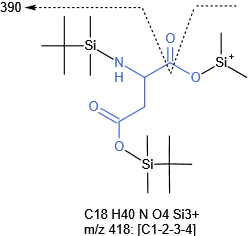 | 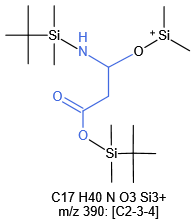 | CO | 1  (Choi et al., 2012)  supplement |
| **Aspartate**  *[418>376]* | 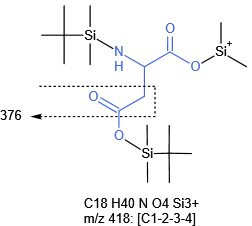 | 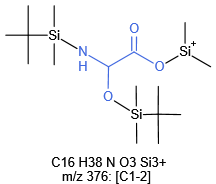 | C2H2O | 1  (Okahashi et al., 2016)  (Choi et al., 2012)  supplement |
| **Aspartate**  *[418>346]* | 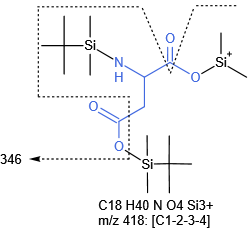 | 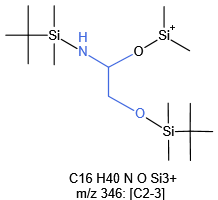 | C2O3 | 1  (Choi et al., 2012) |
| **Aspartate**  *[418>316]* | 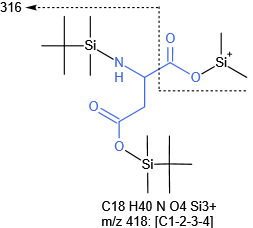 | 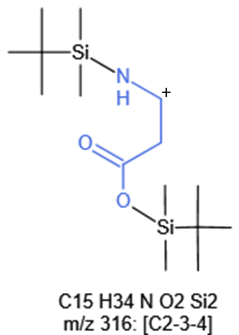 | C3H6O2Si | 1  (Choi et al., 2012)  supplement |
| **Aspartate**  *[418>244]* | 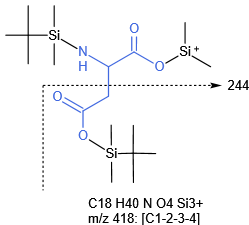 | 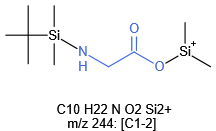 | C8H18O2Si | 1  (Choi et al., 2012)  (Okahashi et al., 2016) |
| **Aspartate**  *[418>216]* | 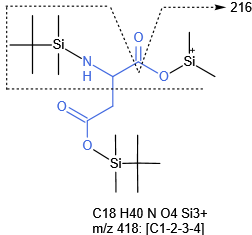 | 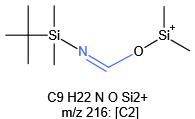 | C9H18O3Si | 2 |
| **Aspartate**  *[418>142]* | 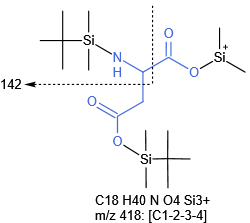 | 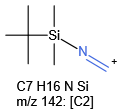 | C11H24O4Si2 | 2 |
| **Aspartate**  *[418>117]* | 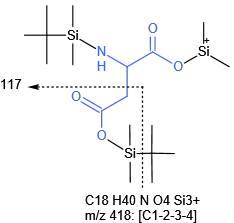 | 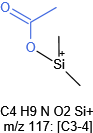 | C14H31NO2Si2 | 1  (Choi et al., 2012)  (Okahashi et al., 2016) |
| **Aspartate**  *[418>103]* | 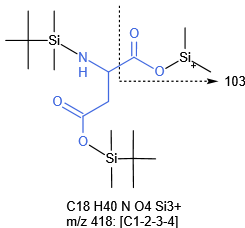 | 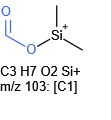 | C15H33NO2Si2 | 1  (Choi et al., 2012) |
| **Aspartate**  *[390>346]* | 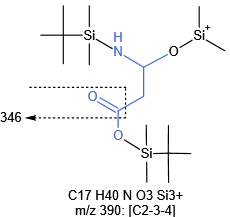 | 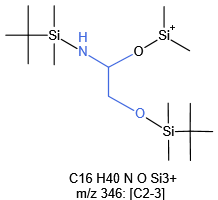 | CO | 1  (Choi et al., 2012)  (Okahashi et al., 2016) |
| **Aspartate**  *[390>216]* | 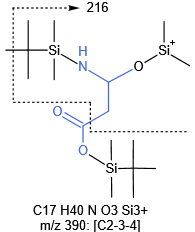 | 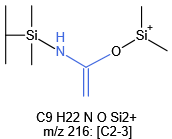 | C8H18O2Si | 1  (Okahashi et al., 2016)  (Choi et al., 2012), supplement |
| **Aspartate**  *[316>218]* | 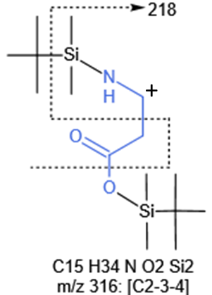 | 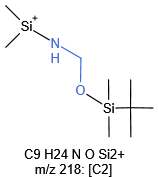 | C6H10O | 2 |
| **Aspartate**  *[316>142]* | 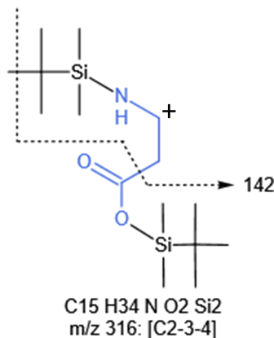 | 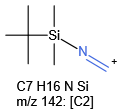 | C8H18O2Si | 2 |

| **Amino acid**  [m/z transition] | **Precursor ion**  (structure, sum formula, m/z) | **Product ion**  (structure, sum formula, m/z) | **Neutral loss** | **Confidence level** |
| --- | --- | --- | --- | --- |
|  |  |  |  |  |
| **Threonine**  *[404>376]* | 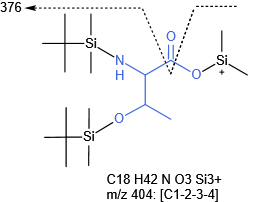 | 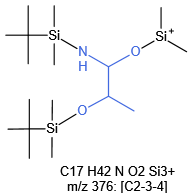 | CO | 2 |
| **Threonine**  *[404>246]* | 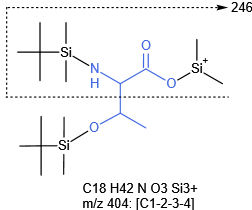 | 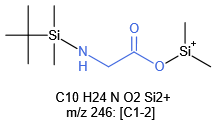 | C8H18OSi | 3 |
| **Threonine**  *[404>244]* | 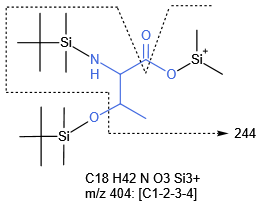 | 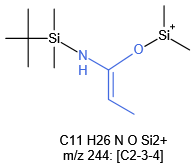 | C7H16O2Si | 2 |
| **Threonine**  *[404>159* | 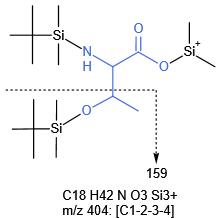 | 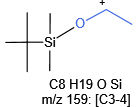 | C10H23NO2Si2 | 3 |
| **Threonine**  *[404>142]* | 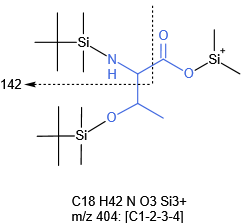 | 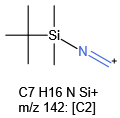 | C11H26O3Si2 | 2 |
| **Threonine**  *[404>103]* | 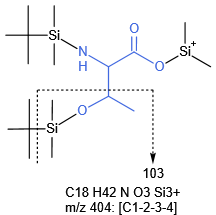 | 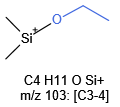 | C14H31NO2Si2 | 3 |
| **Threonine**  *[302>218]* | 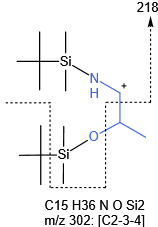 | 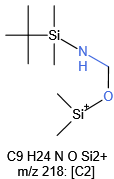 | C6H12O | 3 |
| **Threonine**  *[302>142]* | 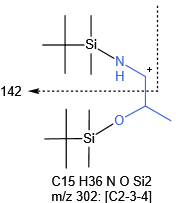 | 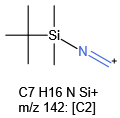 | C8H20O2Si | 2 |

| **Amino acid**  [m/z transition] | **Precursor ion**  (structure, sum formula, m/z) | **Product ion**  (structure, sum formula, m/z) | **Neutral loss** | **Confidence level** |
| --- | --- | --- | --- | --- |
|  |  |  |  |  |
| **Glutamate**  *[474>170]* | 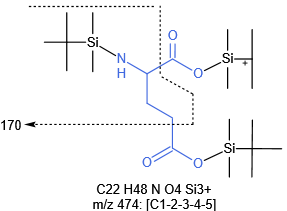 | 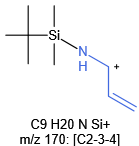 | C13H28O4Si2 | 2 |
| **Glutamate**  *[432>244]* | 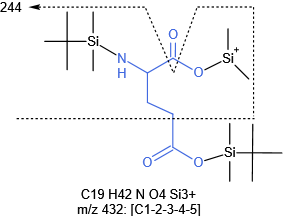 | 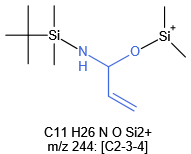 | C8H16O3Si | 2 |
| **Glutamate**  *[432>170]* | 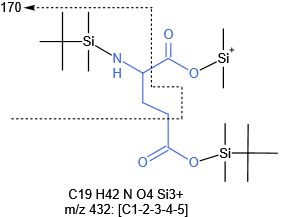 | 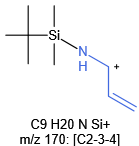 | C10H22O4Si2 | 2 |
| **Glutamate**  *[404>244]* | 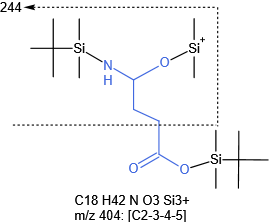 | 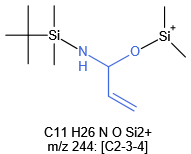 | C7H16O2Si | 1  (Okahashi et al., 2016) |
| **Glutamate**  *[404>170]* | 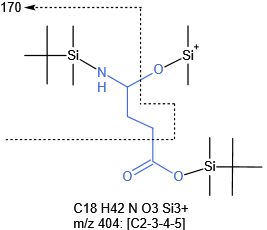 | 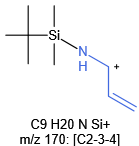 | C9H22O3Si2 | 1  (Okahashi et al., 2016) |
| **Glutamate**  *[330>170]* | 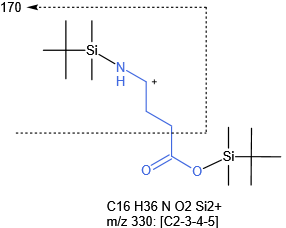 | 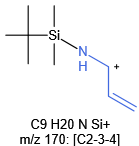 | C7H16O2Si | 1  (Okahashi et al., 2016) |

| **Amino acid**  [m/z transition] | **Precursor ion**  (structure, sum formula, m/z) | **Product ion**  (structure, sum formula, m/z) | **Neutral loss** | **Confidence level** |
| --- | --- | --- | --- | --- |
|  |  |  |  |  |
| **Proline**  *[328>300]* | 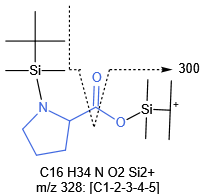 | 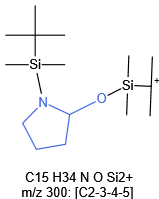 | CO | 2 |
| **Proline**  *[328>168]* | 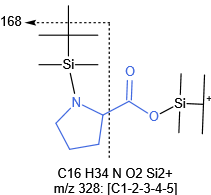 | 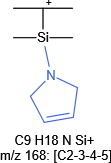 | C6H16O2Si | 2 |
| **Proline**  *[286>258]* | 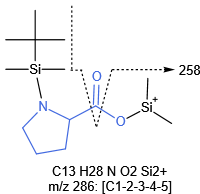 | 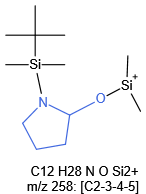 | CO | 2 |
| **Proline**  *[286>184]* | 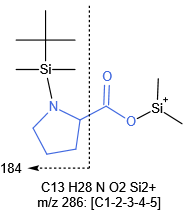 | 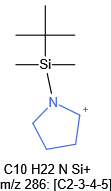 | C3H6O2Si | 2 |

| **Amino acid**  [m/z transition] | **Precursor ion**  (structure, sum formula, m/z) | **Product ion**  (structure, sum formula, m/z) | **Neutral loss** | **Confidence level** |
| --- | --- | --- | --- | --- |
|  |  |  |  |  |
| **Valine**  *[288>260]* | 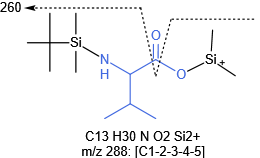 | 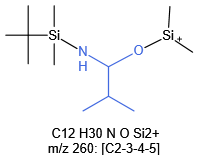 | CO | 2 |
| **Valine**  *[288>216]* | 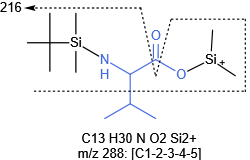 | 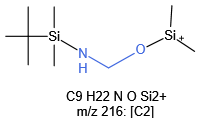 | C4H8O | 2 |
| **Valine**  *[288>186]* | 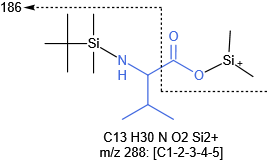 | 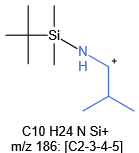 | C3H6O2Si | 2 |
| **Valine**  *[288>103]* | 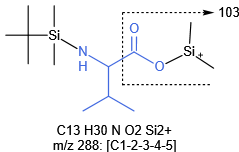 | 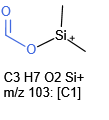 | C10H23NSi | 2 |
| **Valine**  *[186>88]* | 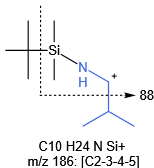 | 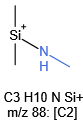 | C7H14 | 2 |

| **Amino acid**  [m/z transition] | **Precursor ion**  (structure, sum formula, m/z) | **Product ion**  (structure, sum formula, m/z) | **Neutral loss** | **Confidence level** |
| --- | --- | --- | --- | --- |
|  |  |  |  |  |
| **Isoleucine**  *[344>316]* | 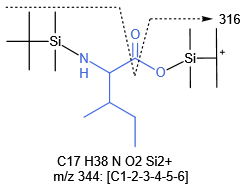 | 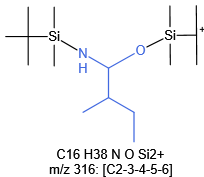 | CO | 2 |
| **Isoleucine**  *[344>200]* |  |  | C6H12O2Si | 2 |
| **Isoleucine**  *[344>184]* |  |  | C7H16O2Si | 2 |
| **Isoleucine**  *[302>274]* |  |  | CO | 2 |
| **Isoleucine**  *[302>200]* |  |  | C3H6O2Si | 2 |
| **Isoleucine**  *[200>142]* |  |  | C4H10 | 2 |
| **Isoleucine**  *[200>88]* |  |  | C8H16 | 2 |

| **Amino acid**  [m/z transition] | **Precursor ion**  (structure, sum formula, m/z) | **Product ion**  (structure, sum formula, m/z) | **Neutral loss** | **Confidence level** |
| --- | --- | --- | --- | --- |
|  |  |  |  |  |
| **Leucine**  *[344>316]* |  |  | CO | 2 |
| **Leucine**  *[344>200]* |  |  | C6H12O2Si | 2 |
| **Leucine**  *[344>103]* |  |  | C14H31NSi | 2 |
| **Leucine**  *[302>274]* |  |  | CO | 2 |
| **Leucine**  *[302>200]* |  |  | C3H6O2Si | 2 |
| **Leucine**  *[302>103]* |  |  | C11H25NSi | 3 |
| **Leucine**  *[274>218]* |  |  | C4H8 | 2 |
| **Leucine**  *[200>158]* |  |  | C3H6 | 2 |
| **Leucine**  *[200>88]* |  |  | C8H16 | 2 |

| **Amino acid**  [m/z transition] | **Precursor ion**  (structure, sum formula, m/z) | **Product ion**  (structure, sum formula, m/z) | **Neutral loss** | **Confidence level** |
| --- | --- | --- | --- | --- |
|  |  |  |  |  |
| **Lysine**  *[431>274]* |  |  | C8H19NSi | 2  (Okahashi et al., 2016) |
| **Lysine**  *[431>272]* |  |  | C7H17NOSi | 2 |
| **Lysine**  *[431>256]* |  |  | \|  \| 1 \| 1 \|  \| 1 \|  \| \| --- \| --- \| --- \| --- \| --- \| --- \|   C8H21NOSi | 2 |
| **Lysine**  *[431>198]* |  |  | C9H23NO2Si2 | 4  (Okahashi et al., 2016) |
| **Lysine**  *[431>144]* |  |  | C13H29NO2Si2 | 3 |
| **Lysine**  *[431>130]* |  |  | C15H35NOSi2 | 2 |

| **Amino acid**  [m/z transition] | **Precursor ion**  (structure, sum formula, m/z) | **Product ion**  (structure, sum formula, m/z) | **Neutral loss** | **Confidence level** |
| --- | --- | --- | --- | --- |
|  |  |  |  |  |
| **Phenylalanine**  *[378>350]* |  |  | CO | 2 |
| **Phenylalanine**  *[378>234]* |  |  | C6H12O2Si | 2 |
| **Phenylalanine**  *[336>308]* |  |  | CO | 2 |
| **Phenylalanine**  *[336>234]* |  |  | C3H6O2Si | 2 |
| **Phenylalanine**  *[336>177]* |  |  | C7H17NOSi | 3 |
| **Phenylalanine**  *[336>91]* |  |  | C10H23NO2Si2 | 2 |
| **Phenylalanine**  *[234>91]* |  |  | C7H17NSi | 2 |

| **Amino acid**  [m/z transition] | **Precursor ion**  (structure, sum formula, m/z) | **Product ion**  (structure, sum formula, m/z) | **Neutral loss** | **Confidence level** |
| --- | --- | --- | --- | --- |
|  |  |  |  |  |
| **Tyrosine**  *[466>438]* |  |  | CO | 2 |
| **Tyrosine**  *[466>364]* |  |  | C3H6O2Si | 2 |
| **Tyrosine**  *[466>307]* |  |  | C7H17NOSi | 2 |
| **Tyrosine**  *[466>221]* |  |  | C10H23NO2Si2 | 2 |

| Fragment  [m/z transition] | **Amino acids** | **Precursor ion**  (structure, sum formula, m/z) | **Product ion**  (structure, sum formula, m/z) | **Neutral loss** | **Confidence level** |
| --- | --- | --- | --- | --- | --- |
| **f302**  *[302>274]* | Ala, Gly, Ile, Ser |  |  | CO | 2 |
| **f302**  *[302>218]* | Ala, Asp, Glu, Gly, Ile, Leu, Lys, Phe, Ser, Thr, Val |  |  | C5H8O | 1  (Okahashi et al., 2016)  Asp, Glu, His, Ile, Leu, Lys, Met, Phe, Ser, Tyr, Val |
| **f302**  *[302>202]* | Ala, Asp, Gly, Pro, Phe, Ser, Tyr |  |  | C6H12O | 2 |
| **f302**  *[302>172]* | Ala, Asp, Gly, Ile, Phe, Pro, Ser, Thr, Val |  |  | C7H18Si | 2 |
| **f302**  *[302>170]* | Gly, Leu, Lys, Pro, Ser, Val |  |  | C6H16OSi | 2 |
| **f302**  *[302>160]* | Ala, Asp, Glu, Gly, Leu, Phe, Pro, Ser, Tyr, Val |  |  | C9H18O | 2 |
| **f302**  *[302>142]* | Ala, Glu, Gly, Ile, Lys, Phe, Pro, Ser, Thr, Tyr, Val |  |  | C7H16O2Si | 2 |

**Confidence levels**

**1:** Confirmed/proven by literature

**2:** Single possible structure, low ppm difference, unequivocal data

**3:** Most likely structure

**4:** Number of backbone carbons experimentally confirmed, inconclusive carbon position
