# Supplementary material for: Improved 13C metabolic flux analysis in Escherichia coli metabolism: application of a high-resolution MS (GC–EI–QTOF) for comprehensive assessment of MS/MS fragments
Source: J Ind Microbiol Biotechnol. 2023 Nov 13;50(1):kuad039. doi: 10.1093/jimb/kuad039 (PMC10716738; doi:10.1093/jimb/kuad039)
Supplement: kuad039_Supplemental_Files [file kuad039_supplemental_files.zip › SupplementaryData A4.docx]

**Supplementary Data A4:** Computation of the number of independent constraints (NIC) for MS & Tandem MS data of proteinogenic amino acids

**Computation of the number of independent constraints (NIC) for:**

**(1) MS data of proteinogenic amino acids**

MS-fragments with equal number of carbon atoms in the amino acid carbon backbone (i.e. redundant fragments such as [M-15]+ & [M-57]+ and [M-85]+ & [M-159]+) do have equal independent constraints. Thus, to compute the number of independent constraints (NIC) for the MS data of each amino acid, only one of the redundant fragments was used, see Table 1.

Due to overlapping fragments, the [f302]+ fragment was not used to calculate the NIC for the amino acids alanine, glycine, leucine, valine and threonine.

**(2) Tandem MS data of proteinogenic amino acids**

Tandem MS-fragments with equal number of carbon atoms in the amino acid carbon backbone of the precursor and product ion do have equal independent constraints. Thus, to compute the number of independent constraints (NIC) for the tandem MS data of each amino acid, only one of the redundant fragments was used, see Table 1.

**Table 1:** MS-fragments and tandem MS-fragments of the proteinogenic amino acids used for NIC computation

| **Amino acid** | **Fragments used for NIC computation** | |
| --- | --- | --- |
|  | MS data | MS/MS data |
| *Glycine* | [M-57]+, [M-85]+ | [246>218], [246>103] |
| *Alanine* | [M-57]+, [M-85]+ | [260>158], [260>103], [f302>142] |
| *Serine* | [M-57]+, [M-85]+, [f302]+ | [390>362], [390>288], [390>142] |
| *Aspartate* | [M-57]+, [M-85]+, [f302]+ | [418>103], [418>244], [418>346], *[418>117], [390>346]* |
| *Threonine* | [M-57]+, [M-85]+ | [404>376], [404>159], [404>142], *[302>142], [404>246], [302>142]* |
| *Glutamate* | [M-57]+, [M-85]+, [f302]+ | [432>244], [404>244], [f302>218] |
| *Proline* | [M-15]+, M159, [f302]+ | [286>258], [f302>202] |
| *Valine* | [M-57]+, [M-85]+, [f302]+ | [288>260], [288>216], [288>103], [186>88], [f302>218] |
| *Isoleucine* | [M-15]+, [M-85]+ | [302>274], [200>88], [f302>218] |
| *Leucine* | [M-15]+, [M-85]+ | [302>274], [302>103], [200>158], [200>88], [f302>218] |
| *Lysine* | [M-15]+, [M-85]+, [f302]+ | [431>274], [431>256], [431>198], [431>144], [431>130], [f302>218] |

To illustrate the applied procedure for NIC computation, an example of computing the number of independent constraints for the tandem MS data of alanine is given below:

**Table 2:** Tandem MS fragments of the proteinogenic amino acid alanine used for NIC computation

| ID | Amino acid | *m/z* | | *Fragment ion* | *Carbon atoms* | |
| --- | --- | --- | --- | --- | --- | --- |
|  |  | *Precursor ion* | *Product ion* | *Precursor ion* | *Precursor ion* | *Product ion* |
| A | Ala | 302 | 232 | [M-15]+ | [1-2-3] | [2-3]* |
| D | Ala | 302 | 103 | [M-15]+ | [1-2-3] | [1]** |
| B | Ala | 260 | 232 | [M-57]+ | [1-2-3] | [2-3]* |
| C | Ala | 260 | 158 | [M-57]+ | [1-2-3] | [2-3]* |
| E | Ala | 260 | 103 | [M-57]+ | [1-2-3] | [1]** |
| F | Ala | 302 | 142 | [f302]+ | [1-2] | [2] |

*: redundant fragments with equal number of carbon atoms in the amino acid carbon backbone of the precursor and product: [1-2-3]>[2-3]; These fragments have equal independent constraints as depicted in Table 3

**: redundant fragments with equal number of carbon atoms in the amino acid carbon backbone of the precursor and product: [1-2-3]>[1]. These fragments have equal independent constraints as depicted in Table 3

**Table 3:** Theoretical tandem mass isotopomer distributions (i.e. matrix N) used for NIC computation

| Alanine isotopomers | | | | | | | | |
| --- | --- | --- | --- | --- | --- | --- | --- | --- |
|  | **A000** | **A100** | **A010** | **A001** | **A101** | **A110** | **A011** | **A111** |
| 302>232 | 72.2 | 0.0 | 0.0 | 0.0 | 0.0 | 0.0 | 0.0 | 0.0 |
| 303>232 | 3.3 | 73.0 | 0.0 | 0.0 | 0.0 | 0.0 | 0.0 | 0.0 |
| 303>233 | 15.6 | 0.0 | 73.0 | 73.0 | 0.0 | 0.0 | 0.0 | 0.0 |
| 304>232 | 0.2 | 2.5 | 0.0 | 0.0 | 0.0 | 0.0 | 0.0 | 0.0 |
| 304>233 | 0.7 | 15.8 | 3.4 | 3.4 | 73.8 | 73.8 | 0.0 | 0.0 |
| 304>234 | 6.4 | 0.0 | 15.0 | 15.0 | 0.0 | 0.0 | 73.8 | 0.0 |
| 305>232 | 0.0 | 0.2 | 0.0 | 0.0 | 0.0 | 0.0 | 0.0 | 0.0 |
| 305>233 | 0.0 | 0.6 | 0.2 | 0.2 | 2.6 | 2.6 | 0.0 | 0.0 |
| 305>234 | 0.3 | 6.5 | 0.7 | 0.7 | 15.2 | 15.2 | 3.4 | 74.6 |
| 305>235 | 0.9 | 0.0 | 6.3 | 6.3 | 0.0 | 0.0 | 14.4 | 0.0 |
| 260>232 | 74.6 | 0.0 | 0.0 | 0.0 | 0.0 | 0.0 | 0.0 | 0.0 |
| 261>232 | 0.9 | 75.4 | 0.0 | 0.0 | 0.0 | 0.0 | 0.0 | 0.0 |
| 261>233 | 16.2 | 0.0 | 75.4 | 75.4 | 0.0 | 0.0 | 0.0 | 0.0 |
| 262>232 | 0.2 | 0.0 | 0.0 | 0.0 | 0.0 | 0.0 | 0.0 | 0.0 |
| 262>233 | 0.2 | 16.4 | 0.9 | 0.9 | 76.3 | 76.3 | 0.0 | 0.0 |
| 262>234 | 6.7 | 0.0 | 15.6 | 15.6 | 0.0 | 0.0 | 76.3 | 0.0 |
| 263>232 | 0.0 | 0.2 | 0.0 | 0.0 | 0.0 | 0.0 | 0.0 | 0.0 |
| 263>233 | 0.0 | 0.0 | 0.2 | 0.2 | 0.0 | 0.0 | 0.0 | 0.0 |
| 263>234 | 0.1 | 6.8 | 0.2 | 0.2 | 15.8 | 15.8 | 0.9 | 77.1 |
| 263>235 | 0.9 | 0.0 | 6.6 | 6.6 | 0.0 | 0.0 | 14.9 | 0.0 |
| 260>158 | 74.6 | 0.0 | 0.0 | 0.0 | 0.0 | 0.0 | 0.0 | 0.0 |
| 261>158 | 6.3 | 75.4 | 0.0 | 0.0 | 0.0 | 0.0 | 0.0 | 0.0 |
| 261>159 | 10.8 | 0.0 | 75.4 | 75.4 | 0.0 | 0.0 | 0.0 | 0.0 |
| 262>158 | 3.0 | 5.5 | 0.0 | 0.0 | 0.0 | 0.0 | 0.0 | 0.0 |
| 262>159 | 0.9 | 11.0 | 6.3 | 6.3 | 76.3 | 76.3 | 0.0 | 0.0 |
| 262>160 | 3.1 | 0.0 | 10.1 | 10.1 | 0.0 | 0.0 | 76.3 | 0.0 |
| 263>158 | 0.1 | 2.9 | 0.0 | 0.0 | 0.0 | 0.0 | 0.0 | 0.0 |
| 263>159 | 0.4 | 0.8 | 3.0 | 3.0 | 5.6 | 5.6 | 0.0 | 0.0 |
| 263>160 | 0.3 | 3.2 | 0.9 | 0.9 | 10.2 | 10.2 | 6.4 | 77.1 |
| 263>161 | 0.3 | 0.0 | 3.1 | 3.1 | 0.0 | 0.0 | 9.4 | 0.0 |
| 260>158 | 74.6 | 0.0 | 0.0 | 0.0 | 0.0 | 0.0 | 0.0 | 0.0 |
| 261>158 | 6.3 | 75.4 | 0.0 | 0.0 | 0.0 | 0.0 | 0.0 | 0.0 |
| 261>159 | 10.8 | 0.0 | 75.4 | 75.4 | 0.0 | 0.0 | 0.0 | 0.0 |
| 262>158 | 3.0 | 5.5 | 0.0 | 0.0 | 0.0 | 0.0 | 0.0 | 0.0 |
| 302>103 | 72.2 | 0.0 | 0.0 | 0.0 | 0.0 | 0.0 | 0.0 | 0.0 |
| 303>103 | 12.9 | 0.0 | 73.0 | 73.0 | 0.0 | 0.0 | 0.0 | 0.0 |
| 303>104 | 6.0 | 73.0 | 0.0 | 0.0 | 0.0 | 0.0 | 0.0 | 0.0 |
| 304>103 | 3.4 | 0.0 | 12.2 | 12.2 | 0.0 | 0.0 | 73.8 | 0.0 |
| 304>104 | 1.1 | 13.1 | 6.1 | 6.1 | 73.8 | 73.8 | 0.0 | 0.0 |
| 304>105 | 2.9 | 5.3 | 0.0 | 0.0 | 0.0 | 0.0 | 0.0 | 0.0 |
| 305>103 | 0.4 | 0.0 | 3.3 | 3.3 | 0.0 | 0.0 | 11.6 | 0.0 |
| 305>104 | 0.3 | 3.5 | 1.0 | 1.0 | 12.4 | 12.4 | 6.2 | 74.6 |
| 305>105 | 0.5 | 1.0 | 2.9 | 2.9 | 5.4 | 5.4 | 0.0 | 0.0 |
| 305>106 | 0.1 | 2.8 | 0.0 | 0.0 | 0.0 | 0.0 | 0.0 | 0.0 |
| 260>103 | 74.6 | 0.0 | 0.0 | 0.0 | 0.0 | 0.0 | 0.0 | 0.0 |
| 261>103 | 10.8 | 0.0 | 75.4 | 75.4 | 0.0 | 0.0 | 0.0 | 0.0 |
| 261>104 | 6.3 | 75.4 | 0.0 | 0.0 | 0.0 | 0.0 | 0.0 | 0.0 |
| 262>103 | 3.1 | 0.0 | 10.1 | 10.1 | 0.0 | 0.0 | 76.3 | 0.0 |
| 262>104 | 0.9 | 10.9 | 6.3 | 6.3 | 76.3 | 76.3 | 0.0 | 0.0 |
| 262>105 | 3.0 | 5.5 | 0.0 | 0.0 | 0.0 | 0.0 | 0.0 | 0.0 |
| 263>103 | 0.3 | 0.0 | 3.1 | 3.1 | 0.0 | 0.0 | 9.4 | 0.0 |
| 263>104 | 0.3 | 3.2 | 0.9 | 0.9 | 10.2 | 10.2 | 6.4 | 77.1 |
| 263>105 | 0.4 | 0.8 | 3.0 | 3.0 | 5.6 | 5.6 | 0.0 | 0.0 |
| 263>106 | 0.1 | 2.9 | 0.0 | 0.0 | 0.0 | 0.0 | 0.0 | 0.0 |
| 302>142 | 72.2 | 0.0 | 0.0 | 72.2 | 0.0 | 0.0 | 0.0 | 0.0 |
| 303>142 | 9.4 | 73.0 | 0.0 | 9.4 | 73.0 | 0.0 | 0.0 | 0.0 |
| 303>143 | 9.6 | 0.0 | 73.0 | 9.6 | 0.0 | 0.0 | 73.0 | 0.0 |
| 304>142 | 3.2 | 8.7 | 0.0 | 3.2 | 8.7 | 0.0 | 0.0 | 0.0 |
| 304>143 | 1.3 | 9.7 | 9.5 | 1.3 | 9.7 | 73.8 | 9.5 | 73.8 |
| 304>144 | 2.9 | 0.0 | 8.9 | 2.9 | 0.0 | 0.0 | 8.9 | 0.0 |

**Table 4:** Number of independent constraints for the tandem MS data of alanine calculated by the following equation: NIC = rank(N), with the matrix N corresponding to the theoretical tandem mass isotopomer distributions for the respective fragments depicted in Table 3.

| **Fragments of matrix N** | **NIC = rank(N)** | **Comment** |
| --- | --- | --- |
| A or B or C | 6 | redundant fragments: [1-2-3]>[2-3] |
| ABC | 6 | redundant fragments: [1-2-3]>[2-3] |
| D or E | 6 | redundant fragments: [1-2-3]>[1] |
| DE | 6 | redundant fragments: [1-2-3]>[1] |
| F | 4 | fragment [f302]+: [1-2]>[2] |
| ADF | 8 |  |
| BEF | 8 |  |
| ABCDEF | 8 |  |

As depicted in Table 4, tandem MS-fragments with equal number of carbon atoms in the amino acid carbon backbone of the precursor and product ion do have equal independent constraints. Thus, to compute the number of independent constraints for the tandem MS data of each amino acid, only one of the redundant fragments needs to be used.
